# Physicochemical Characterization of Functional Lignin–Silica Hybrid Fillers for Potential Application in Abrasive Tools

**DOI:** 10.3390/ma9070517

**Published:** 2016-06-25

**Authors:** Beata Strzemiecka, Łukasz Klapiszewski, Artur Jamrozik, Tadeusz J. Szalaty, Danuta Matykiewicz, Tomasz Sterzyński, Adam Voelkel, Teofil Jesionowski

**Affiliations:** 1 Faculty of Chemical Technology, Institute of Chemical Technology and Engineering, Poznan University of Technology, Berdychowo 4, Poznan PL-60965, Poland; lukasz.klapiszewski@put.poznan.pl (Ł.K.); artur.robert.jamrozik@gmail.com (A.J.); tadeusz.h.szalaty@doctorate.put.poznan.pl (T.J.S.); adam.voelkel@put.poznan.pl (A.V.); 2 Faculty of Construction Engineering and Management, Institute of Materials Technology, Poznan University of Technology, Piotrowo 3, Poznan PL-61138, Poland; danuta.matykiewicz@put.poznan.pl (D.M.); tomasz.sterzynski@put.poznan.pl (T.S.)

**Keywords:** lignin–silica fillers, lignin, phenolic resins, thermo-mechanical and physicochemical properties, abrasive tools

## Abstract

Functional lignin–SiO_2_ hybrid fillers were prepared for potential application in binders for phenolic resins, and their chemical structure was characterized. The properties of these fillers and of composites obtained from them with phenolic resin were compared with those of systems with lignin or silica alone. The chemical structure of the materials was investigated by Fourier transform infrared spectroscopy (FT-IR) and carbon-13 nuclear magnetic resonance spectroscopy (^13^C CP MAS NMR). The thermal stability of the new functional fillers was examined by thermogravimetric analysis–mass spectrometry (TG-MS). Thermo-mechanical properties of the lignin–silica hybrids and resin systems were investigated by dynamic mechanical thermal analysis (DMTA). The DMTA results showed that abrasive composites with lignin–SiO_2_ fillers have better thermo-mechanical properties than systems with silica alone. Thus, fillers based on lignin might provide new, promising properties for the abrasive industry, combining the good properties of lignin as a plasticizer and of silica as a filler improving mechanical properties.

## 1. Introduction

Interface interactions in composites are crucial for their final properties. Control over the surface and interface aspects of polymers and their fillers are of paramount importance. Robust interfaces are critical regions that not only enable the long-term use of the polymer composites, but also contribute to the performance of the end products. For this reason, they must be controlled at the molecular level with appropriate chemistry strategies to ensure their long-term stability. One of the important objectives of this research is to control the surface composition and reactivity of the inorganic material embedded in the polymer matrices. For this reason, intensive research is being conducted on hybrid materials: organic-inorganic compounds with relevance to many composites (e.g., structural adhesives, hairy nanoparticles for optical sensors, medical implants, etc.), as well as abrasive tools. The rationale for the application of hybrid materials is two-fold: (i) to improve the dispersion of nanofillers in polymer matrices [1]; and (ii) to enhance the mechanical properties of the host polymer matrix [1,2].

Brostow et al. [3] proposed a relationship between toughness and brittleness that can be very valuable in relation to expectations for outcomes of material design modifications. Thus, the effect of reinforcement of polymers by a filler or functionalized filler can be evaluated quantitatively.

Djouani et al. [1] demonstrated by dynamic mechanical analysis (DMA), performed on the reinforced epoxy matrix, that introducing a small quantity of clay/PGMA nanofillers into an amine-cured epoxy (from 1 to 5 wt. %) led to significant increase in the storage modulus and a drop in the loss factor tan *δ*.

Recently, a thorough study by Matyjaszewski et al. [4] highlighted the benefit of employing polymer-grafted filler particles in terms of transparency, a sign of excellent dispersion of the polymer-grafted filler in the polymer matrix.

Silica-recycled wood sawdust was described by Brostow et al. [5] and showed better thermal stability, better compatibility with the polymer matrix and higher values of E’ in DMA studies. We have described an earlier application of lignin–silica hybrids to polypropylene and have shown that the addition of silica to lignin via this new hybrid formulation has a positive effect on the thermal stability of PP/silica-lignin composites [6].

The functional hybrid fillers presented in this paper may have potential applications in abrasive articles. One of the traditional formulations consists of a wetting agent (resole, also acting as a binder), a binder (novolac), micrometer-sized abrasive particles (e.g., alumina–corundum) and commonly known fillers (e.g., clay, zeolite, and carbon black). Fillers play a very important role in abrasive articles for example, they protect the resin from decomposition [7,8,9].

More than a century after their invention, phenolic resins are among the most versatile resin systems, with annual production of ca. 5 million tons (data from 2008) [10], rising on average by 3% annually. Due to the very good binding properties of this type of material, phenolic resins have been used primarily in the production of laminate flooring, chipboard and grinding materials. In abrasives, they are used as binders, due to their hardness, chemical and thermal resistance and favorable cost-performance characteristics [10,11]. The requirements set for abrasive tool binders are typically very high, because they are subject to extreme conditions such as significantly elevated temperatures and heavy mechanical loads, and are exposed to many types of industrial coolants.

The abrasive tool industry uses two types of phenol-formaldehyde resins: (i) resole, a liquid thermosetting resin, containing up to 20% of free phenol which is released gradually during resin storage, processing and application of the finished products; used to wet the surface of the abrasive grain; and (ii) novolac, a solid thermoplastic resin, used mostly with the addition of 8%–12% hexamine as a curing agent during the hardening process hexamine decomposes into formaldehyde and ammonia.

Binders obtained from phenolic resins offer high efficiency of grinding, exceptional durability and flexibility, and are less prone to scorch the surface. Notable disadvantages of these binders include their sensitivity to base-containing coolants at high temperature, and susceptibility to the occurrence of microvoids [12]. Their major drawback, however, is the emission of harmful compounds, especially phenol and formaldehyde, during storage of the substrates, production, and use of the final products. Secretion of free phenol and formaldehyde from both prepolymers and cured resins has an adverse effect on the environment and on humans. It also causes an unpleasant odor during processing and throughout the use of the phenol-containing products. Novolac resin is cured using hexamine, which during the curing process at 180 °C decomposes into ammonia and formaldehyde, compounds that are highly toxic and malodorous.

The main aim of this work is the synthesis and comprehensive characterization of innovative lignin–silica hybrid fillers as well as the thermo-mechanical properties of model abrasive articles with these new fillers, having different lignin-to-silica ratios. It can be expected that the application of such hybrid fillers in abrasive articles may: (i) eliminate the use of hexamethylenetetramine (hexa) and/or phenol compounds by replacing the content of resole and/or hexa by lignin; and (ii) reinforce the effect obtained by application of silica. Lignin can also have a positive effect on the viscoelastic properties of the obtained composites [13,14,15,16]. Thus, such hybrid fillers may be very promising, pro-ecological fillers for the abrasive industry. Moreover, this represents a new way of taking advantage of lignin—a renewable resource whose recycling is highly desirable.

## 2. Results

### 2.1. FT-IR Spectroscopy

**Figure 1:**
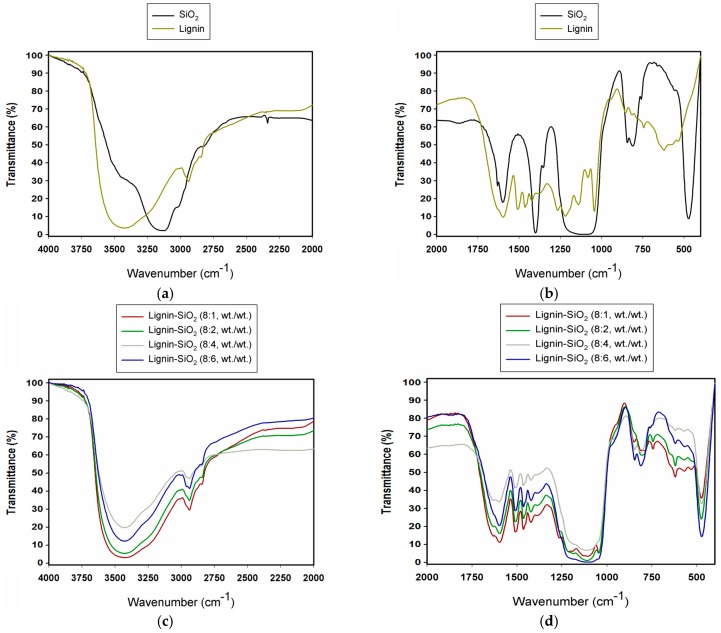
FT-IR spectra of: the precursors silica and lignin (**a**,**b**); and lignin–silica hybrid materials (**c**,**d**).

The FT-IR spectra of both lignin and silica are shown in Figure 1a,b, and FT-IR spectra of lignin–silica hybrid fillers are presented in Figure 1c,d. The most important bands are summarized in Table 1.

The FT-IR analysis helps to detect characteristic functional groups of the precursors and of the resulting hybrid materials. The following bands were detected for lignin: stretching vibrations of aromatic hydroxyl groups at 3426 cm^−1^, C−H stretching vibrations from methyl and methylene residue (2940 cm^−1^), C=O stretching vibrations at 1637 cm^−1^ attributed to carbonyl groups, and characteristic vibrations from aromatic rings at 1600 cm^−1^, 1509 cm^−1^ and 1421 cm^−1^. At the wavenumbers 1271 cm^−1^, 1143 cm^−1^, 856 cm^−1^, and 744 cm^−1^ were absorption maxima characteristic of the guaiacyl unit in lignin. The spectrum of the biopolymer also reflects stretching vibrations of C–O–C etheric bonds (1045 cm^−1^). The spectrum obtained for lignin is consistent with published data [17,18,19].

For SiO_2_, signals were observed at 3142 cm^−1^ attributed to stretching vibrations of O−H groups, and at 1107 cm^−1^, which is characteristic for silica (Si−O−Si). Signals at 845 cm^−1^ and 812 cm^−1^ correspond to asymmetric and symmetric stretching vibrations of Si–O bonds, and their bending mode appeared at 473 cm^−1^. These data are in agreement with available literature data for silica [17,18,19,20].

Figure 1b shows the spectra of selected lignin–silica hybrid materials. These contain characteristic bands of lignin and silica in accordance with the previously described spectra of the precursors. As the quantity of lignin increased, the intensity of its bands was stronger. Moreover, no signal is observed for the O−H group from SiO_2_, which means that these O–H groups might react with lignin. A mechanism for the interaction of those components was proposed in [21]. It is concluded that the process was successfully completed with satisfactory results.

### 2.2. ^13^C CP MAS NMR Spectroscopy

**Figure 2:**
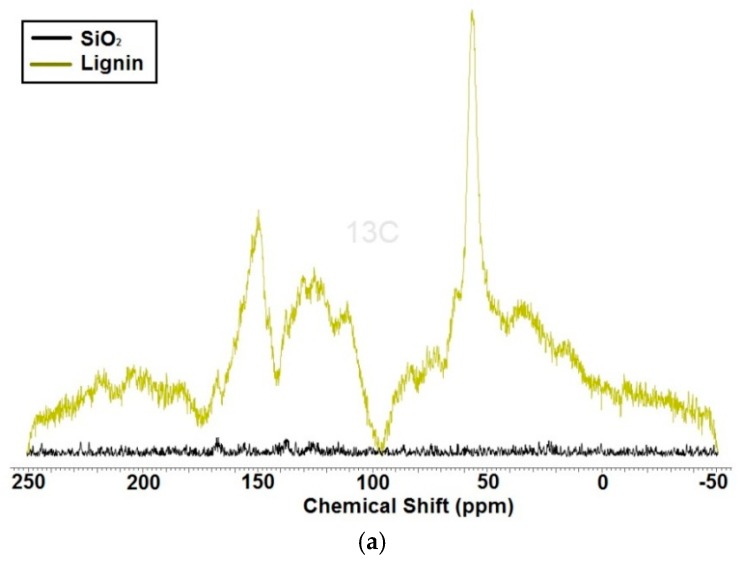

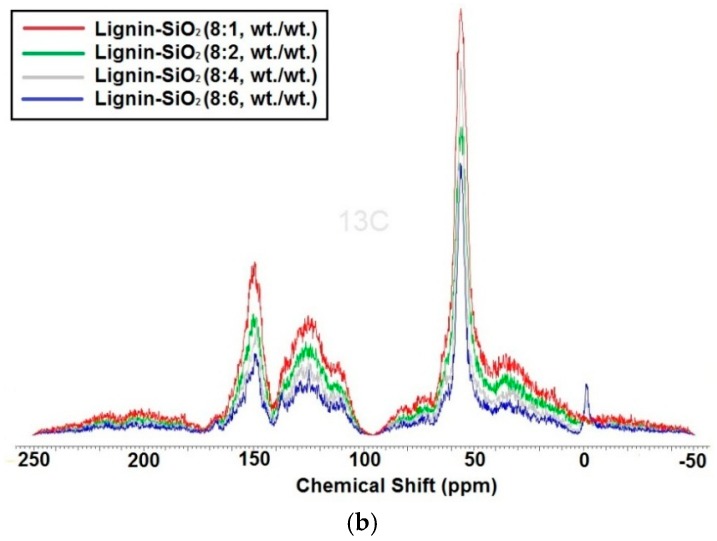
^13^C CP MAS NMR spectra of: lignin and silica (**a**); and lignin–silica hybrid fillers (**b**).

In Figure 2a, the ^13^C CP MAS NMR spectra of lignin and SiO_2_ are presented. It is clear from the lack of signals in the NMR spectrum that the SiO_2_ is not contaminated by organic compounds. The spectrum of kraft lignin exhibits characteristic signals for that material [22,23]. Detailed results of carbon–13 nuclear magnetic resonance analysis are presented in Table 2. Comparing the chemical shifts for lignin with those for lignin–SiO_2_ hybrids, it can be noted that some of them decrease. On the other hand, some of them remained strong, for instance the signal from the aromatic carbon atom with methoxy group (56.0 ppm), 5–5 non-etherified bands (125.0 ppm) and ether linkage from syringyl units (150.5 ppm). Intermolecular interactions between silica and lignin caused the intensity of some characteristic chemical shifts for the biopolymer to be reduced. This is observed especially for C atoms near the ether bond: C–*γ* in G type *β*–O–4 units, C–*γ* in *β*–*β*, C–*γ* in *β*–aryl ether, C–2/C–6 in H units. Thus, it can be expected that these units interact with SiO_2_.

Lignin has a complicated structure and low degree of symmetry, so it produced a large quantity of signals in the NMR spectra. For that reason the change in signal intensity was small and the effect of the increased amount of silica was not significant.

In conclusion, analysis of the ^13^C CP MAS NMR spectra fully confirmed that the synthesis of the lignin–silica hybrid filler had been performed successfully.

### 2.3. TG-MS Analysis

**Figure 3:**
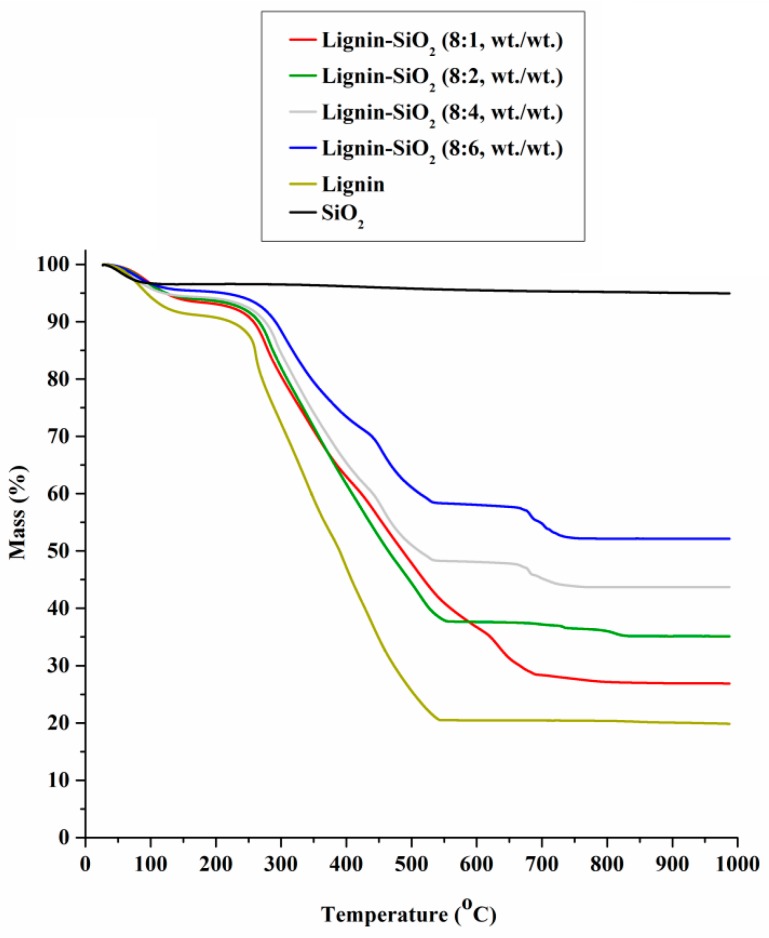
Thermogravimetric curves for lignin, silica and lignin–silica hybrid fillers.

The effectiveness of preparation of the lignin–SiO_2_ hybrid fillers was verified by the TG-MS method. Thermogravimetric curves for lignin, silica and the organic–inorganic hybrids are presented in Figure 3.

The TG curve recorded for SiO_2_ indicates a small mass loss of about 4%, confirming the high thermal stability of the silica. The small mass loss is linked to the presence of crystalline water in the SiO_2_ structure. Physically bound water had been removed, since the silica used in the study had previously undergone a drying process (105 °C, 4 h) [6,18,24]. The TG curve for lignin indicates a significant mass loss of about 69% relative to the initial mass of the sample [6,18,25,26,27,28]. The first small mass loss of about 16%, taking place up to 230 °C, is mainly a result of the removal of water bound on the lignin surface. In that temperature range, there also occurs the degradation of the propanoid side chain of the biopolymer. The second, larger mass loss of about 48% taking place in the range 230–530 °C is related to the complex thermal decomposition of lignin (especially the *β*–*β* and C−C bonds between lignin basic units), involving the formation of new bonds as a consequence of crosslinking reactions. The third mass loss of about 5% observed in the range 530–1000 °C is interpreted as a consequence of fragmentation of the molecules in uncontrolled and undetermined reactions [27]. The TG curves presented here are in agreement with [5]. Emitted compounds are listed in Table 3. Generally, they are phenol derivatives. The results suggest that in lignin, mainly weak aliphatic bonds are broken with increasing temperature. The detected compounds listed in Table 3 imply that the lignin used is rich in syringyl units [28]. It can be noted that, compared with lignin, additional compounds are emitted from the lignin–SiO_2_ fillers, such as 3-methylfuran, 1,3-cyclohexadiene and benzene. This demonstrates that lignin and SiO_2_ react during the formation of the hybrid.

The study also included preliminary tests of emissions of harmful compounds into the atmosphere. The samples analyzed were 8:1 and 8:6 lignin:silica with resole and novolac, and the reference sample. The quantity of phenol emitted into the atmosphere was assessed using the head-space method. The phenol adsorbed on SDB sorbent was desorbed by methanol and then analyzed by high-performance liquid chromatography HPLC. The eluent was water:methanol (20:80, v/v). The column was silica–C18, length 200 mm. For the reference sample the emitted amount of phenol from 1 g of sample was about 80 mg during 2 h, while the quantity was 60 mg for the 8:1 sample and 40 mg for the sample with 8:6 lignin to silica.

### 2.4. Dynamic-Mechanical Properties

**Figure 4:**
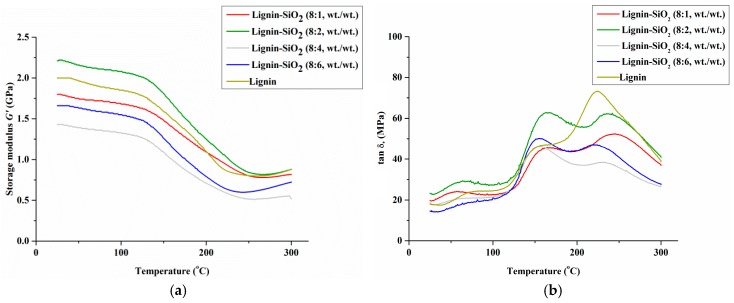
Dynamic mechanical storage modulus G’ (**a**) and tan *δ* (**b**) versus temperature for composites consisting of 80% abrasive grains and with filler (lignin, silica or lignin–silica hybrids).

The influence of the lignin fillers on the viscoelastic behavior of phenolic resin was described in terms of storage modulus G′ and glass transition temperature Tg [29]. Comprehensive analysis of DMTA results provides information about stiffness, degree of crosslinking and interfacial bonding between filler and matrix [30]. Plots of the storage modulus G’ and mechanical loss factor (tan *δ*) versus temperature T are presented in Figure 4a,b. The values of the composite G’ at various temperatures and glass transition temperature Tg are given in Table 4. For a sample containing only SiO_2_ a homogeneous stiff material was not obtained; therefore it was not possible to investigate its thermo-mechanical properties.

The highest value of G’ (2.21 GPa at 25 °C and 2.16 GPa at 50 °C) out of all modified samples was recorded for the samples of lignin–SiO_2_ in a ratio of 8:2 (wt./wt.). The lowest value of G’ (1.43 GPa at 25°C) was recorded for the lignin–SiO_2_ (8:4, wt./wt.) sample, which may be caused by inadequate miscibility between filler and matrix in this composite. Moreover, Tg values for the samples containing 1 and 2 parts by weight of silica were significantly higher than for the reference sample. This confirms that silica particles in combination with lignin can be used as a reinforcement agent for phenolic resin. In this case the Tg values increased from 206 °C to 250 °C and 252 °C. These effects may be the result of such factors as increased rigidity of the polymer chain, reduced free volume, and increased crosslinking density [31]. The glass transition temperature value is relative to chain flexibility and the free volume associated with the chemical structure of composites [32,33]. Lignin exhibits a complicated spatial chemical structure. As a consequence, its presence in the phenol matrix may result in increased distance between the polymer chains and reduction in the Tg value, as was observed for the lignin sample. It should be noted that combining lignin particles with porous silica particles having high surface area led to a rigid hybrid material. In this case, it was assumed that the introduction of rigid functional lignin–SiO_2_ hybrid fillers into the phenol matrix restricts the movement of polymer chains and increases the Tg of the composites [34].

In both of these cases (lignin–SiO_2_ 8:1, wt./wt. and 8:2, wt./wt.) an increased crosslinking density can be observed as a result of interaction between lignin and silica particles. Lignin presents a high quantity of activated free ring positions, which may react with other compounds [35]. The high reactivity of the hybrid fillers can be associated with a high crosslinking density. The presence of rigid functional groups can restrict the distance between nodes in the polymer network and thus increase the crosslinking density [21,36].

On the tan *δ* curves, the first peak centered at 150–200 °C was observed. It may be ascribed to the glass transition temperature of the uncured phenol resin [37] or to wood lignin, which is softened in this temperature range when plasticized [38,39]. The second significant peak on the tan *δ* curves describes the glass transition temperature of the composites where the network’s mechanical properties are subject to change [38]. The width of the tan *δ* curve for the modified composites was greater than for the reference sample. Significantly, the increased width demonstrates the improved ability of the composites to dissipate energy through molecular motion [40]. In the case of the other samples, the G′ and Tg values of the composites decreased with an increase in silica content. In Figure 4a an increase in the storage modulus above 250 °C is observed for the lignin–SiO_2_ (8:6, wt./wt.) sample. This phenomenon may be caused by inadequate miscibility between filler and matrix in this composite. On heating to temperatures above Tg (230 °C for lignin–SiO_2_ (8:6, wt./wt.) samples) the materials change their properties, and the increasing values of the storage modulus may result from measurement error or partial degradation of the material.

A high quantity of silica and lignin molecules can hinder the complete curing of the resin matrix [41]. This can be explained by the penetration of the filler particles between the polymer chains and the fact that the active groups can be separated. The reference sample was prepared without silica filler, and has the lowest glass transition value, which may result from the plasticizing properties of lignin.

Summarizing, the analysis confirms that lignin filler can react with silica particles, and the resulting rigid structures can be used as reinforcement for polymer composites.

It should be mentioned here that interesting fillers for the abrasive industry may also include graphite oxide, which increases the Tg of epoxy resin, probably by catalyzing the crosslinking reaction of this resin, and graphene oxide [42,43,44]. It would appear to be of interest to combine graphene oxide with lignin to produce a multifunctional filler. Such research will be undertaken in the future.

## 3. Materials and Methods

### 3.1. Preparation of Lignin–SiO_2_ Hybrid Materials

The lignin–silica hybrid materials were prepared by a mechanical method. More details of this method of obtaining organic-inorganic hybrids can be found in previously published work [16]. In the present study, the commercial silica Syloid^®^244 (WR Grace & Co., Columbia, MD, USA) and kraft lignin (Sigma-Aldrich, Munich, Germany) were used. The final products in the form of lignin–SiO_2_ hybrids were produced using 8 parts by weight of lignin with 1, 2, 4 and 6 parts of silica. Silica and lignin were ground and simultaneously mixed using a Pulverisette 6 Classic Line planetary ball mill (Fritsch, Dresden, Germany). To obtain a suitably homogeneous final material, grinding lasted 6 h. To prevent possible overheating of the material due to continuous grinding, every two hours the mill automatically switched off for 5 min, after which it began operating again. Immediately after grinding, the lignin–silica hybrid material was sifted using a sieve with a mesh diameter of 40 μm.

### 3.2. Preparation of Abrasive Composites with Lignin–SiO_2_ Hybrids and with Lignin and SiO_2_

The model abrasive composites for DMTA studies were prepared by mixing resole, filler, novolac and abrasive grains, in ratios of 3:5:12:80 by weight. The proportions of the components were chosen as the standard values used in the abrasive industry. The components were mixed using a mechanical mixer at a slow rate of 200 rpm for a short time (about 3 min). The water content of the resole was ca. 10 wt. %. White fused alumina with a 120 mesh granulation was used as an abrasive. Novolac contains 12% of hexamethylenetetramine (hexamine). Firstly, the abrasive grains were covered by resole, and then the mixture of novolac and filler was added and homogenized. The composites prepared in this way were formed into cuboids for DMTA studies (see Section 3.4). 

Thus, the following composites were studied by DMTA, denoted for simplicity as follows:

- SiO_2_ means 3 wt. % resole + 80 wt. % white fused alumina + 12 wt. % novolac + 5 wt. % SiO_2_;
- lignin means 3 wt. % resole + 80 wt. % white fused alumina + 12 wt. % novolac + 5 wt. % lignin;
- lignin–SiO_2_ (8:1, wt./wt.) means 3 wt. % resole + 80 wt. % white fused alumina + 12 wt. % novolac + 5 wt. % lignin–silica;
- lignin–SiO_2_ (8:2, wt./wt.) means 3 wt. % resole + 80 wt. % white fused alumina + 12 wt. % novolac + 5 wt. % lignin–silica;
- lignin–SiO_2_ (8:4, wt./wt.) means 3 wt. % resole + 80 wt. % white fused alumina + 12 wt. % novolac + 5 wt. % lignin–silica;
- lignin–SiO_2_ (8:6, wt./wt.) means 3 wt. % resole + 80 wt. % white fused alumina + 12 wt. % novolac + 5 wt. % lignin–silica.

The samples were then hardened according to following temperature program: heating from 50 °C up to 180 °C; heating rate 0.2 °C/min; then heating at 180 °C for 10 h.

### 3.3. Physicochemical Evaluation

#### 3.3.1. FT-IR Spectroscopy

The presence of the expected functional groups was confirmed by Fourier transform infrared (FT-IR) spectroscopy, using a Vertex 70 spectrophotometer (Bruker, Karlsruhe, Germany). The materials were analyzed in the form of tablets, made by placing a mixture of anhydrous KBr (ca. 0.25 g) and 1.5 mg of the tested substance in a steel ring under a pressure of 10 MPa. The tests were performed at a resolution of 0.5 cm^−1^ in the wavenumber range 4000–400 cm^−1^.

#### 3.3.2. ^13^C CP MAS NMR Spectroscopy

^13^C CP MAS NMR measurement was carried out on a DSX spectrometer (Bruker, Karlsruhe, Germany). For the determination of NMR spectra, a sample of about 100 mg was placed in a ZrO_2_ rotator with diameter 4 mm, which enabled spinning of the sample. Centrifugation at the magic angle was performed at a spinning frequency of 8 kHz. The ^13^C CP MAS NMR spectra were recorded at 100.63 MHz in a standard 4 mm MAS probe using a single pulse excitation with high power proton decoupling (pulse repetition 10 s, spinning speed 8 kHz).

#### 3.3.3. TG-MS Analysis

TG-MS curves were obtained using a thermogravimetric analyzer coupled to the MS (1 PYRIS TGA/MS CLARUS 680 SQ8, Perkin Elmer, Waltham, MA, USA). The apparatus uses electron streams (EI) with energy 70 eV. The measurements were performed in a helium atmosphere (gas flow 40 mL/min) at a heating rate of 20 °C/min. The samples were heated up to 1000 °C, starting from 30 °C.

### 3.4. Dynamic-Mechanical Properties

The dynamic-mechanical properties of cured samples with dimensions of 10 mm × 4 mm × 50 mm were investigated by DMTA measurements in torsion mode using an Anton Paar MCR 301 apparatus (Anton Paar GmbH, Buchs, Switzerland) operating at frequency f = 1 Hz over a temperature range between 25 °C and 300 °C at a heating rate of 2 °C/min. The position of tan *δ* at its maximum was taken as the glass transition temperature.

## 4. Conclusions

The results presented in this paper demonstrate that lignin–SiO_2_ hybrid fillers can be obtained in a relatively simple way by intensive mechanical mixing of silica with lignin. Both ^13^C CP MASS NMR and FT-IR results showed that in these conditions lignin can form covalent chemical bonds with SiO_2_—probably silanol groups in the silica structure react with lignin. The functional hybrid fillers are thermally stable, as was proved by TG analysis. TG-MS analysis showed that the lignin–SiO_2_ hybrids decompose to different compounds than lignin. Moreover, DMTA showed that the thermo-mechanical properties of the model abrasive composite depend on the lignin-to-silica ratio in the hybrid filler. The best thermo-mechanical properties were obtained for a lignin–SiO_2_ filler with a ratio of lignin to silica of 8:2. The sample with SiO_2_ did not form a homogeneous hard material and it was not possible to investigate its thermo-mechanical properties. Thus, it is not possible to use pure silica as a filler in an abrasive tool.

In summary, the use of lignin–SiO_2_ filler can give a final product with good thermo-mechanical properties, and such products would emit smaller quantities of harmful compounds into the atmosphere.

## Figures and Tables

**Table 1:** Vibrational frequency wavenumbers (cm^−1^) observed for lignin, silica and lignin–silica hybrid fillers.

Kraft Lignin (cm^−1^)	Silica (cm^−1^)	Lignin–Silica Hybrid Filler (cm^−1^)	Vibrational Assignment
3426	3142	3434	O−H stretching
2940	–	2940	CH_x_ stretching
1637	–	1637	C=O stretching
–	1602	1601	H_2_O (physically adsorbed water)
1600	–	1600	C−C, C=C (aromatic skeleton), stretching
1509	–	1508	
1465	–	1467	C−H (CH_3_ + CH_2_), bending
1421	–	1424	C−C, C=C (aromatic skeleton), stretching
1271	–	1271	C−O (guaiacyl unit) stretching
1219	–	1222	C−OH (phenolic OH) stretching
1143	–	1142	Aromatic C−H (guaiacyl unit), stretching
–	1107	1110	Si−O−Si stretching
1080	–	1085	C−O stretching
1045	–	1044	C−OH + C−O−C (aliphatic OH + ether) stretching
856	–	–	Aromatic C−H (guaiacyl unit), bending
–	845	845	Si−O asymmetric stretching
–	812	809	Si−O symmetric stretching
744	–	746	Aromatic C−H (guaiacyl unit), bending
536	–	531	CH_x_ bending
–	473	476	Si−O bending

**Table 2:** Chemical shifts (ppm) observed for lignin, silica and lignin–silica hybrid fillers.

Kraft Lignin (ppm)	Lignin–Silica Hybrid Filler (ppm)	Assignment
14.2	14.3	*γ*–CH_3_ in n-propyl side chain
35.3	34.0	CH_3_ group, ketones (conj.) or in aliphatic
54.0	55.2	C–*β* in *β*–*β* and *β*–5 units
55.6	56.0	C in Ar–OCH_3_
62.0	62.0	C–*γ* in G type *β*–1 and *β*–5 units
64.3	–	C–*γ* in G type *β*–O–4 units with *α*–C=O
71.1	71.0	C–*α* in G type *β*–O–4 units (threo)
72.0	72.0	C–*α* in G type *β*–O–4 units (erythro)
72.5	–	C–*γ* in *β*–*β*; C–*γ*, *β*–aryl ether
83.0	82.9	C–*β* in guaiacyl type *β*–O–4 units (erythro)
85.1	–	C–*β* in guaiacyl type *β*–O–4 units (threo)
110.5	111.0	C–2 in guaiacyl units
112.0	–	C–2 in G units
115.1	–	C–5 in G units
118.7	119.0	C–6 in G units
122.0	–	C–1 and C–6 in Ar–C(=O)C–C units
125.2	125.0	C–5, non-etherified 5–5
128.0	–	C–2/C–6, in H units
131.2	–	C–1, non-etherified 5–5
132.3	–	C–5, etherified 5–5
133.2	133.0	C–1 in non-etherified G and S units
138.1	137.5	C–4, syringyl etherified
148.2	148.8	C–3, guaiacyl units
151.2	150.5	C–3/C–5, etherified S units
169.5	–	C=O in *φ*–COOH, ester C=O in *φ*–C(=O)OR and R–C(=O)OCH_3_

**Table 3:** Compounds detected in TG-MS analysis for lignin–silica hybrid fillers and pure lignin.

Compound	Lignin–SiO_2_ (8:1, wt./wt.)	Lignin–SiO_2_ (8:2, wt./wt.)	Lignin–SiO_2_ (8:4, wt./wt.)	Lignin–SiO_2_ (8:6, wt./wt.)	Lignin
Carbon dioxide	+	+	+	+	+
Sulfur dioxide	+	+	+	+	+
Methanethiol	+	+	+	+	+
Acetone	+	+	+	+	+
Dimethyl sulfide	+	+	+	+	+
Acetic acid	+	+	+	+	+
Furan, 3-methyl	+	+	+	−	−
1,3-cyclohexadiene	+	+	+	−	−
2-propanone, 1-hydroxy	+	+	+	+	+
Benzene	+	+	+	+	-
Phenol	+	+	+	+	+
Phenol, 2-methyl	+	+	+	+	+
P-cresol	+	+	+	+	+
Phenol, 2-methoxy	+	+	+	+	+
Benzene, 1,2-dimethoxy	+	+	+	+	+
Phenol, 2,4-dimethyl	+	+	+	+	+
Phenol, 2,3-dimethyl	+	+	+	+	+
Phenol, 4-ethyl	+	+	+	+	+
Cresol	+	+	+	+	+
Benzene, 1-ethyl-2-methoxy	+	−	−	−	−
Benzene, 4-ethyl-2-methoxy	+	+	+	−	+
2-methoxy-4-vinylphenol	+	−	−	−	+
Vanillin	+	+	+	−	+

**Table 4:** Values of storage modulus G’ and glass transition temperature Tg for composites based on lignin–silica hybrids and pure lignin.

Sample	G’ 25 °C (GPa)	G’ 50 °C (GPa)	G’ 300 °C (GPa)	tan *δ_max_*	T_g_ (°C)
Lignin–SiO_2_ (8:1, wt./wt.)	1.80	1.74	0.82	0.0654	252
Lignin–SiO_2_ (8:2, wt./wt.)	2.21	2.16	0.88	0.0718	250
Lignin–SiO_2_ (8:4, wt./wt.)	1.43	1.39	0.52	0.0708	220
Lignin–SiO_2_ (8:6, wt./wt.)	1.66	1.62	0.73	0.0748	230
Lignin	2.00	1.95	0.88	0.0859	206

## References

[1] Djouani F., Herbst F., Chehimi M.M., Benzarti K. (2011). Synthesis, characterization and reinforcing properties of novel, reactive clay/poly(glycidyl methacrylate) nanocomposites. Constr. Build. Mater..

[2] Solhia L., Ataia M., Nodehia A., Imania M., Ghaemib A., Khosravi K. (2012). Poly(acrylic acid) grafted montmorillonite as novel fillers for dental adhesives: Synthesis, characterization and properties of the adhesive. Dental Mater..

[3] Brostow W., Hagg Lobland H.E., Khoja S. (2015). Brittleness and toughness of polymers and other materials. Matter. Lett..

[4] Dang A., Ojha S., Hui C.M., Mahoney C., Matyjaszewski K., Bockstaller M.R. (2014). High-transparency polymer nanocomposites enabled by polymer-graft modification of particle fillers. Langmuir.

[5] Brostow W., Datashvili T., Jiang P., Miller H. (2016). Recycled HDPE reinforced with sol–gel silica modified wood sawdust. Eur. Polym. J..

[6] Klapiszewski L., Bula K., Sobczak M., Jesionowski T. (2016). Influence of processing conditions on the thermal stability and mechanical properties of PP/silica-lignin composites. Int. J. Polym. Sci..

[7] Strzemiecka B., Voelkel A., Hinz M., Rogozik M. (2014). Application of inverse gas chromatography in physicochemical characterization of phenolic resin adhesives. J. Chromatogr. A.

[8] Strzemiecka B., Voelkel A., Chmielewska D., Sterzyński T. (2014). Influence of different fillers on phenolic resin abrasive composites. Comparison of inverse gas chromatographic and dynamic mechanical–thermal analysis characteristics. Int. J. Adhes. Adhes..

[9] Strzemiecka B., Voelkel A., Donate-Robles J., Martín-Martínez J.M. (2013). Estimation of polyurethane-carbon black interactions by means of inverse gas chromatography. J. Chromatogr. A.

[10] Weissig V., Pettinger T.K., Murdock N. (2014). Nanopharmaceuticals (Part 1): Products on the market. Int. J. Nanomed..

[11] Such G.K., Johnston A.P.R., Liang K., Caruso F. (2012). Synthesis and functionalization of nanoengineered materials using click chemistry. Prog. Polym. Sci..

[12] Wong K.V., Perilla N., Paddon A. (2013). Nanoscience and nanotechnology in solar cells. J. Energy Resour. Technol..

[13] Com M., Lligadas G., Ronda J.C., Gali M., Cadiz V. (2013). Renewable benzoxazine monomers from “lignin-like” naturally occurring phenolic derivatives. J. Polym. Sci..

[14] Tejado A., Kortaberria G., Pena C., Labidi J., Echeverría J.M., Mondragon I. (2007). Lignins for phenol replacement in novolac-type phenolic formulations, Part I: Lignophenolic resins synthesis and characterization. J. Appl. Polym. Sci..

[15] Basso M.C., Giovando S., Pizzi A., Celzard A., Fierro V. (2013). Tannin/furanic foams without blowing agents and formaldehyde. Ind. Crop. Prod..

[16] Li X., Pizzi A., Lacoste C., Fierro V., Celzard A. (2013). Tannin-furan foams & blowing agent. Bioresources.

[17] Klapiszewski Ł., Nowacka M., Milczarek G., Jesionowski T. (2013). Physicochemical and electrokinetic properties of silica/lignin biocomposites. Carbohydr. Polym..

[18] Bula K., Klapiszewski Ł., Jesionowski T. (2015). A novel functional silica/lignin hybrid material as a potential bio-based polypropylene filler. Polym. Compos..

[19] Grząbka-Zasadzińska A., Klapiszewski Ł., Bula K., Jesionowski T., Borysiak S. (2016). Supermolecular structure and nucleation ability of polylactide-based composites with silica/lignin hybrid fillers. J. Therm. Anal. Calorim..

[20] Al-Oweini R., El-Rassy H. (2009). Synthesis and characterization by FTIR spectroscopy of silica aerogels prepared using several Si(OR)_4_ and R’’Si(OR’)_3_ precursors. J. Mol. Struct..

[21] Strzemiecka B., Klapiszewski Ł., Matykiewicz D., Voelkel A., Jesionowski T. (2016). Functional lignin-SiO_2_ hybrids as potential fillers for phenolic binders. J. Adhes. Sci. Technol..

[22] Wen J., Sun S., Xue B., Sun R. (2013). Recent advances in characterization of lignin polymer by solution-state nuclear magnetic resonance (NMR) methodology. Materials.

[23] Wysokowski M., Klapiszewski Ł., Moszyński D., Bartczak P., Szatkowski T., Majchrzak I., Siwińska-Stefańska K., Bezhenov V.V., Jesionowski T. (2014). Modification of chitin with kraft lignin and development of new biosorbents for removal of cadmium(II) and nickel(II) ions. Mar. Drugs.

[24] Klapiszewski Ł., Królak M., Jesionowski T. (2014). Silica synthesis by the sol-gel method and its use in the preparation of multifunctional biocomposites. Cent. Eur. J. Chem..

[25] Kijima M., Hirukawa T., Hanawa F., Hata T. (2011). Thermal conversion of alkaline lignin and its structured derivatives to porous carbonized materials. Bioresour. Technol..

[26] Brebu M., Cazacu G., Chirila O. (2011). Pyrolysis of lignin—A potential method for obtaining chemicals and/or fuels. Cellulose Chem. Technol..

[27] Brebu M., Vasile C. (2010). Thermal degradation of lignin—A review. Cellulose Chem. Technol..

[28] Brebu M., Tamminen T., Spiridon I. (2013). Thermal degradation of various lignins by TG-MS/FTIR and Py-GC-MS. J. Anal. Appl. Pyrol..

[29] Menard K.P., Brostow W. (2000). Thermal transitions and their measurement. Performance of Plastics.

[30] Candan Z., Gardner D.J., Shaler S.M. (2016). Dynamic mechanical thermal analysis (DMTA) of cellulose nanofibril/nanoclay/pMDI nanocomposites. Compos. B Eng..

[31] Chandran M.S., Sanil K., Sunithaa K., Mathewa D., Raoa V.L., Naira C.P.R. (2016). Alder-ene polymers derived from allyl aralkyl phenolic resin and bismaleimides: Carbon fiber composites properties. Polym. Adv. Technol..

[32] Eesaee M., Shojaei A. (2014). Effect of nanoclays on the mechanical properties and durability of novolac phenolic resin/woven glass fiber composite at various chemical environments. Compos. Part A.

[33] Sawpan M.A., Holdsworth P.G., Renshaw P. (2012). Glass transitions of hygrothermal aged pultruded glass fibre reinforced polymer rebar by dynamic mechanical thermal analysis. Mater. Des..

[34] Chiang C.L., Ma C.C.M. (2004). Synthesis, characterization, thermal properties and flame retardance of novel phenolic resin/silica nanocomposites. Polym. Degrad. Stabil..

[35] Tejado A., Peña C., Labidi J., Echeverria J.M., Mondragon I. (2007). Physico-chemical characterization of lignins from different sources for use in phenol–formaldehyde resin synthesis. Bioresour. Technol..

[36] Benyahya S., Aouf C., Caillol S., Boutevin B., Pascault J.P., Fulcrand H. (2014). Functionalized green tea tannins as phenolic prepolymers for bio-based epoxy resins. Ind. Crop. Prod..

[37] Park B.D., Wang X.M. (2005). Thermokinetic behavior of powdered phenol-formaldehyde (PPF) resins. Thermochim. Acta.

[38] Kelley S.S., Rials T.G., Glasser W.G. (1987). Relaxation behaviour of the amorphous components of wood. J. Mater. Sci..

[39] Wang J., Laborie M.P.G., Wolcott M.P. (2009). Kinetic analysis of phenol–formaldehyde bonded wood joints with dynamical mechanical analysis. Thermochim. Acta.

[40] Santhosh Kumar K.S., Reghunadhan Nair C.P., Ninan K.N. (2008). Silica fiber–polybenzoxazine–syntactic foams; Processing and properties. J. Appl. Polym. Sci..

[41] Alonso M.V., Oliet M., García J., Rodríguez F., Echeverría J. (2007). Master curve and time–temperature–transformation cure diagram of lignin–phenolic and phenolic resol resins. J. Appl. Polym. Sci..

[42] Mauro M., Acocella M.R., Corcione C.E., Maffezzoli A., Guerra G. (2014). Catalytic activity of graphite-based nanofillers on cure reaction of epoxy resins. Polymer.

[43] Giuri A., Rella S., Malitesta C., Colella S., Listorti A., Gigli G., Rizzo A., Cozzoli P.D., Acocella M.R., Guerra G. (2015). Synthesis of reduced graphite oxide by a novel green process based on UV light irradiation. Sci. Adv. Mater..

[44] Rella S., Giuri A., Corcione C.E., Acocella M.R., Colella S., Guerra G., Listorti A., Rizzo A., Malitesta C. (2015). X-ray photoelectron spectroscopy of reduced graphene oxide prepared by a novel green method. Vacuum.

